# Electrocardiogram and left atrial abnormality: Design of an observational study to clarify diagnostic criteria

**DOI:** 10.1111/anec.12770

**Published:** 2020-05-29

**Authors:** Juan Lacalzada‐Almeida, María Manuela Izquierdo‐Gómez, Ignacio Laynez‐Cerdeña, Javier García‐Niebla, Vanesa Bruña, Antonio Bayés de Luna, Manuel Martínez‐Sellés

**Affiliations:** ^1^ Department of Cardiology Hospital Universitario de Canarias Tenerife Spain; ^2^ Valle del Golfo Health Center Servicio Canario de Salud El Hierro Spain; ^3^ Department of Cardiology Hospital General Universitario Gregorio Marañón CIBERCV Madrid Spain; ^4^ Fundació Investigació Cardiovascular ICCC Barcelona Spain; ^5^ Universidad Europea Universidad Complutense Madrid Madrid Spain

**Keywords:** electrocardiogram, interatrial block, left atrial abnormality, speckle tracking echocardiography

## Abstract

**Background:**

The criteria applied for diagnosis of left atrial (LA) abnormality using electrocardiogram (ECG) have high specificity but low sensitivity. In fact, some authors have suggested classifying P‐wave anomalies associated with LA abnormality and interatrial block as “atrial abnormalities.” The most widely known ECG criteria for LA abnormality include P‐wave duration, morphology and voltage of P wave in inferior leads, presence of P‐wave terminal force in V_1_ (PtfV_1_), and P‐wave axis and area. PtfV_1_ has also been reported to vary according to misplacement of the V_1_ and V_2_ electrodes.

**Methods:**

The objective of this observational cohort study is to determine the degree of correlation between ECG criteria for LA abnormality and left atrium volume and functionality, as determined by speckle tracking echocardiography. The study also aims to investigate the correlation between these echocardiographic parameters and PtfV_1_ value by placing the V_1_ and V_2_ electrodes in the second, third, and fourth intercostal spaces.

**Results and Conclusions:**

Our results could help to clarify whether the decrease in left atrial deformity, which is currently considered a surrogate target of fibrosis, correlates better with ECG criteria for LA abnormality than atrial volumes.

## 1 INTRODUCTION

The criteria applied for diagnosis of left atrial (LA) abnormality using electrocardiogram (ECG) are based on various parameters including P‐wave duration ≥ 120 ms, the classic Morris index (increase in P‐wave terminal force in V_1_ [PtfV_1_], with terminal negativity of P wave in V_1_ < −0.1 mV and duration >0.04 s) (Morris, Estes, Whalen, & Thompson, 1964), P‐wave morphology in inferior leads, P voltage in lead I, P‐wave area/axis, and P‐wave score(Alexander et al., 2019). These criteria have high specificity (85%–90%) but low sensitivity (Josephson, Kastor, & Morganroth, 1977; Tsao et al., 2008; Bayes de Luna, 2012) and can also be used for diagnosis of interatrial block (IAB). Therefore, a consensus statement of the American Heart Association suggests including these P‐wave changes, whether due to LA abnormality or IAB, as "P‐wave abnormalities" (Hancock et al., 2009). Furthermore, young athletes might have LA abnormality with P‐wave duration < 120 ms (Hock et al., 2019). The Morris index (Morris et al., 1964), which is the only criterion with acceptable sensitivity, was recently shown to be unreliable (Sajeev et al., 2019). Furthermore, the negativity of the P wave increases (Rasmussen et al., 2019) if the V_1_ electrode is placed in the second intercostal space. Some authors have suggested that this V_1_ pattern represents no more than an interatrial conduction defect and may not necessarily indicate LA abnormality (Josephson et al., 1977).

The main aim of our observational study is to compare the association between P‐wave criteria in patients with LA abnormality on transthoracic echocardiography (TTE). As secondary objectives, we aim to study the association between P‐wave criteria in patients with left atrium (LA) functional abnormalities on speckle tracking echocardiography (STE). In addition, we will analyze the correlation between these echocardiographic parameters and PtfV_1_ value by placing the V_1_ and V_2_ electrodes in the second, third, and fourth intercostal spaces.

## 2 METHODS

### 2.1 Design and study population

**Figure 1:**
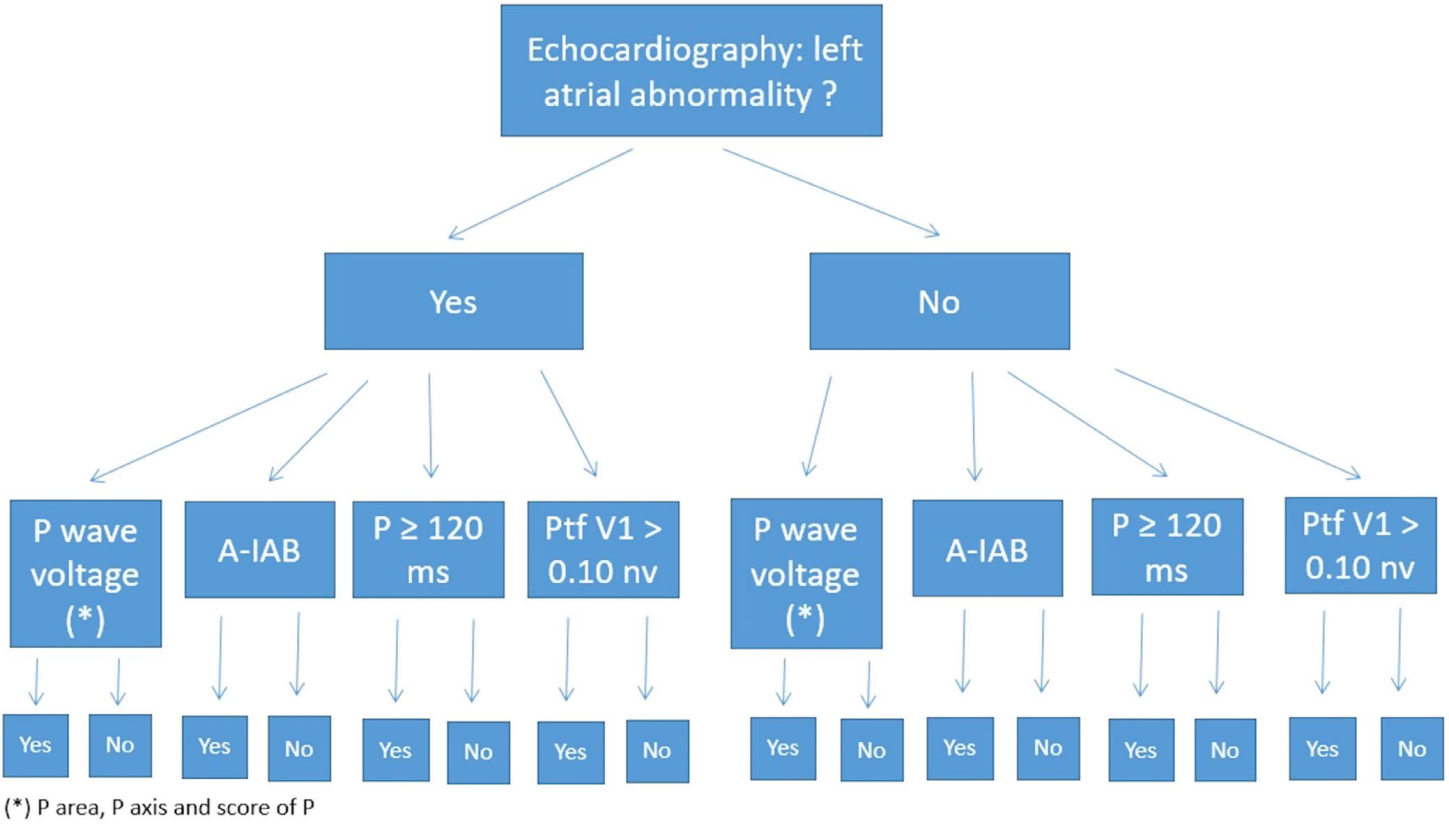
Study design. A‐IAB, advanced interatrial block; PtfV1, P terminal force V1

We will perform a prospective observational cohort study in the Echocardiography and Non‐invasive Cardiology Laboratory of the Hospital Universitario de Canarias. The study population will comprise patients referred for TTE (Figure 1).

### 2.2 Inclusion criteria

- Age ≥ 60 years.
- Signed informed consent.
- Optimal‐quality ECG and echocardiography images.

### 2.3 Exclusion criteria

- Poor transthoracic window.
- Atrial fibrillation (all types).
- Previous arrhythmias (atrial and ventricular).
- Absence of measurable P waves.
- Cardiac devices.
- Chest abnormalities (including pectus excavatum and straight back syndrome).
- Chronic obstructive pulmonary disease.

### 2.4 P‐wave parameters

**Figure 2:**
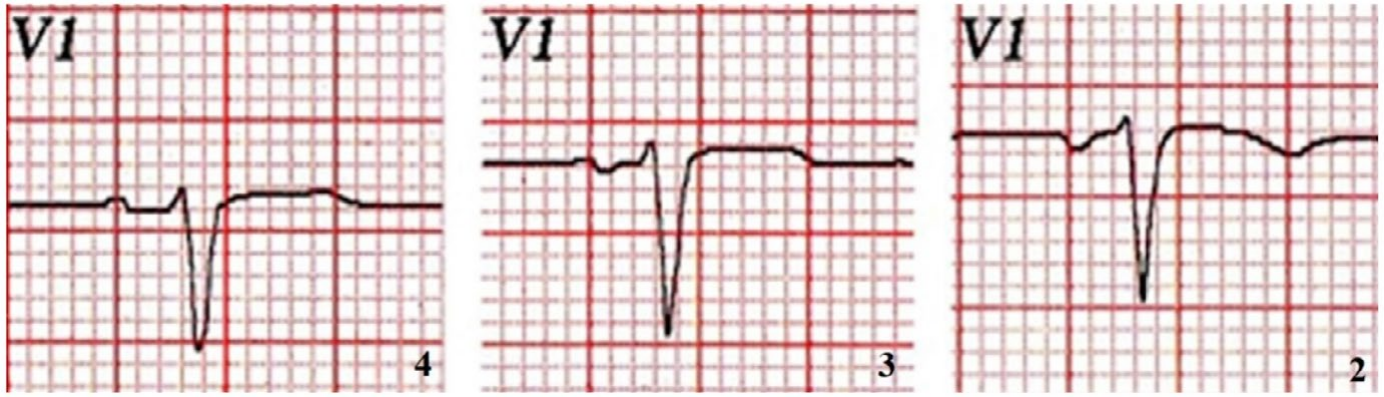
V1 lead of a healthy patient with normal left atrial size for echocardiography, with the V1 electrode located in the correct place: fourth intercostal space (4), third intercostal space (3), and second intercostal space (2). Note how a normal P wave becomes progressively negative as the V1 electrode is located upward

The ECGs will be obtained according to the standards set at 25 mm/s and 10 mm/mv, with a filtering range of 0.05–150 Hz and AC filter at 50 Hz. In addition, the branch position of the V_1_ and V_2_ electrodes will be intentionally varied in 3 successive ECGs (position in the fourth, third, and second intercostal space; Figure 2)

. The ECGs will be analyzed by 2 experienced cardiologists (VB and MMS), who will be blinded to the TTE and STE data. The P‐wave duration will be measured in the frontal plane leads in digital ECG (amplified 20 times) using GeoGebra 4.2 software. The interval between the earliest and the latest detection of atrial depolarization will be used. The following ECG parameters will be included to evaluate LA and right atrial (RA) abnormalities (Alexander et al., 2019; Hancock et al., 2009; Kaplan, Evans, Foster, Lim, & Schiller, 1994; Lee et al., 2007; Tsao et al., 2008).

ECG parameters for assessment of LA abnormality:

1. P‐wave duration (<120 ms, 120–140 ms, >140 ms)
2. P‐wave morphology in the inferior leads (nonbiphasic or biphasic)
3. Presence of PtfV_1_
4. P voltage in lead I (>0.20 mV, 0.10–0.20 mV, <0.10 mV)
5. P‐wave area and axis
6. P‐wave MVP score
7. Length of the P wave in lead II > 120 ms
8. Left deviation of the middle axis of the P wave between −30 and −45º.

The ECG criteria for diagnosing RA abnormality are as follows:

1. P‐wave amplitude in lead II > 2.5 mm.
2. Upward deflection of the P wave in lead V1 > 1.5 mm in amplitude.
3. Increase of the area under the initial positive part of the P wave in V1 > 0.06 mm‐s.
4. Right deviation of the middle axis of the P wave greater than +75º.

### 2.5 Echocardiography studies

Echocardiography studies will be stored digitally and will be analyzed by 2 experienced operators (JLA and MMIG), who will be blinded to the clinical ECG data. The operators will use a commercial system (iE33 xMATRIX, Koninklijke Philips NV) with a 2–4 MHz multifrequency transducer. Three consecutive beats will be recorded in a cineloop format during apnea. The analysis will be performed using an echocardiographic analysis system (Xcelera R2, Philips). LA and left ventricular (LV) measurements and LV ejection fraction and Doppler variables will be measured to quantify LV diastolic function according to standard echocardiographic methods (Nishimura & Tajik, 1997) (Table 1). The maximal LA volume (LAV max) will be traced in both the apical 4‐ and 2‐chamber views in the “frame” prior to mitral valve opening using the biplane disk summation algorithm to calculate its volume, adjusted for body surface area and considering an LAmax > 34 ml/m^2^ as increased (Lang et al., 2015). The size of the RA will be quantified using the apical four‐chamber view, and the volume will be determined using the disk summation technique in 2D mode (Lang, et al., 2015).

**Table 1:** Doppler echocardiography with atrial strain variables

LAmin, BS (ml/m^2^)
LAmax, BS (ml/m^2^)
LAVpre, BS (ml/m^2^)
Anteroposterior diameter measurement of left atrium in long axis parasternal in 2D, mm
LAEF, %
LAEF passive, %
LAEF active, %
LASr, %
LAScd, %
LASct, %
pLASRr, 1/s
pLASRcd, 1/s
pLASRct, 1/s
Pulsed mitral Doppler: E wave, cm/sec
Pulsed mitral Doppler: A wave, cm/sec
E/A ratio
E‐wave deceleration time, msec
A‐wave duration, msec
Pulsed Doppler pulmonary vein PVs, cm/sec
Pulsed Doppler pulmonary vein PVd, cm/sec
S/D ratio
Mitral annular tissue Doppler: e´ septal, cm/sec
Mitral annular tissue Doppler: a´, septal, cm/sec
Mitral annular tissue Doppler: s´, septal, cm/sec
Mitral annular tissue Doppler: e´, lateral, cm/sec
Mitral annular tissue Doppler: a´, lateral, cm/sec
Mitral annular tissue Doppler: s´, lateral, cm/sec
E/e´ ratio
Vp, cm/sec
E mitral/Vp ratio.
LVED dimension, mm
LVES dimension, mm
LVED volume index, ml/m^2^
LVES volume index, ml/m^2^
Relative wall thickness
Left ventricular mass index, g/m^2^
Left ventricular ejection fraction, biplane (%)
RA volume (ml/m^2^)
RASr, %
RAScd, %
RASct, %
pRASRr, 1/s
pRASRcd, 1/s
pRASRct, 1/s

Abbreviations: BS, body surface; LAEF, total left atrial emptying fraction; LAmax, maximum volume of left atrium; LAmin, minimum volume of left atrium; LAScd, left atrial strain during conduit phase; LASct, left atrial strain during contraction phase; LASr, left atrial strain during reservoir phase; LAVpre, left atrium volume at the beginning of the P wave of the ECG; LVED, left ventricular end‐diastolic; LVES, left ventricular end‐systolic; pLASRcd, left atrial peak strain rate during conduct phase; pLASRct, left atrial peak strain rate during contraction phase; pLASRr, left atrial peak strain rate during reservoir phase; pRASRcd, (negative) peak strain rate during conduit phase; pRASRct, (negative) peak strain rate during contraction phase; pRASRr, (positive) peak strain rate during reservoir phase; PVd, pulsed Doppler pulmonary vein, diastole pulmonary vein; PVs, pulsed Doppler pulmonary vein, systole pulmonary vein; RA, right atrial; RAScd, strain during conduit phase; RASct, strain during contraction phase; RASr, strain during reservoir phase; Vp, M mode color, left intraventricular propagation velocity.

For the STE of the LA, standard 2D images from apical 4‐ and 2‐chamber views will be acquired, with a narrow sector angle (30°–60°) and a frame rate of 60–90 frames per second (Badano et al., 2018). The LA endocardial border will be traced manually in both 4‐ and 2‐chamber views by marking 2 points at both ends of the mitral annulus and a third at the ceiling of the LA in end systole. The surface epicardial tracing is automatically generated by the system and can be adjusted manually by the operator in cases of tracking failure. Any segments that subsequently fail to track will be excluded. The LA myocardium will be divided into 12 segments of interest. For analysis, a longitudinal strain and strain rate (SR) will be measured automatically “offline” (Yasuda et al., 2015) using QLAB Advanced Tissue Motion Quantification (Philips) Release 8.1 equipped with STE analysis software. Global longitudinal strain and SR will be the average of the 12 values obtained for each LA segment (Table 1). Zero strain reference will be set at LV end diastole or when the LA speckle tracking is triggered on QRS onset (Badano et al., 2018)^,^(Olsen et al., 2016). The periods of the cardiac cycle will be determined after obtaining the LA longitudinal strain/SR and aligning them with the pulsed Doppler spectrum of the LV inflow and outflow tracts, starting at QRS onset (Badano et al., 2018)^,^(Olsen et al., 2016). RA deformation will be assessed in the apical four‐chamber view in 2D by starting the tracing at the endocardial border of the RA in the tricuspid annulus and continuing along the lateral wall of the RA, roof of the RA, and septal wall of the RA, before finishing at the opposite border of the tricuspid annulus. The apical four‐chamber view will be optimized by avoiding RA foreshortening, thus enabling—as with the LA—the volumes, strain, and strain rate of the RA to be determined (Badano et al., 2018).

### 2.6 Other variables

Sociodemographic variables, presence of comorbidities, pharmacological treatments, and indications for echocardiography will be recorded (Table 2).

**Table 2:** Demographic variables and indications for echocardiography study

Demographic variables
Age
Sex
Height, cm
Weight, kg
BMI, kg/m^2^
BSA, m^2^
Diabetes mellitus
Smoking
Hypertension
Hyperlipidemia
TIA or previous stroke
Previous diagnosis of heart failure
Known coronary artery disease
Obstructive sleep apnea
CHADS_2_ score
CHA2DS₂‐VASc score
ACE inhibitors/ARBs
Beta blockers
Anticoagulants or NOACs
Antiplatelet agents
Antiarrhythmic agents
Indications for echocardiography study
Chest pain
Coronary artery disease
Valvulopathy
Cardiomyopathy
LVEF calculation
Dyspnea
Murmur
Syncope
Hypertension
Rule out structural heart disease
Pulmonary hypertension
Aortic aneurysm

Abbreviations: ACE, angiotensin‐converting enzyme; ARBs, angiotensin II receptor blockers; BMI, body mass index; BSA, body surface area; LVEF, left ventricular ejection fraction; NOACs, new oral anticoagulant agents; TIA, transitory ischemic attack.

### 2.7 Sample size calculation

Previous data suggested that 92% of patients with LA abnormality might have a positive Morris index (Morris et al., 1964). We estimate that 79 patients with a positive Morris index are required to determine this rate with a confidence level of 95% and precision error of 6%.

### 2.8 Statistical analysis

Continuous variables will be compared using the *t* test or the Mann–Whitney test. Categorical variables will be compared using the chi‐square test. Based on the volume of LAV max by echocardiography, 2 groups will be established, as follows: increased volume and normal volume. Correlations between ECG and TTE STE variables will be obtained using the Spearman rank test and the intraclass correlation coefficient. We will use the logistic regression model adjusted for potential confounders, including variables that are significant in the univariate model. The ability of the multivariate logistic model to correlate with the LAV max group will be verified using receiver operating characteristic (ROC) curves. The overall accuracy, sensitivity, specificity, and positive and negative predictive values for the optimal cutoff will be calculated using the Youden index. Intra‐ and interobserver variability will be assessed using Bland–Altman analysis. All statistical analyses will be performed using IBM SPSS Statistics for Windows, Version 23 (IBM Corp., Armonk, NY, USA).

### 2.9 Ethical considerations

This study will be reviewed and approved by the Clinical Research Ethics Committee of Hospital Universitario de Canarias. All participants will be informed about the aims and procedures of the project and will sign a written informed consent document. The project will be conducted in accordance with the World Medical Association Declaration of Helsinki related to Ethical Principles for Medical Research Involving Human Subjects, the Convention on Human Rights and Biomedicine of the Council of Europe (1997), and the Additional Protocol to the Convention on Human Rights and Biomedicine, concerning Biomedical Research (2005).

### 2.10 Study timeline

The study is expected to begin in June 2020. The estimated end of the inclusion period is July 2020, and the estimated completion date for the study is September 2020.

## 3 CONCLUSION

Our prospective observational study will help to define ECG criteria for LA and RA abnormality and to distinguish IAB from LA abnormality.

## CONFLICTS OF INTEREST

The authors declare no potential conflicts of interest.

## AUTHOR CONTRIBUTIONS

MMS, ABL, and JLA involved in conception and design of the study. MMS, ABL, MMIG, and JLA involved in drafting of the case description and critical revision of the study. MMS, IL, VB, JGN, and JLA involved in methodological and statistical design of the study. All authors were involved at each stage of the revision process and contributed substantially to the project's intellectual content.
